# Association of Pediatric Inpatient Socioeconomic Status With Hospital Efficiency and Financial Balance

**DOI:** 10.1001/jamanetworkopen.2019.13656

**Published:** 2019-10-18

**Authors:** Morgane Michel, Corinne Alberti, Jean-Claude Carel, Karine Chevreul

**Affiliations:** 1 Unité de Recherche Clinique en Économie de la Santé Eco Ile de France, Hôtel Dieu, Assistance Publique-Hôpitaux de Paris, Paris, France; 2 Unité d'Epidémiologie Clinique, Assistance Publique-Hôpitaux de Paris, Hôpital Robert Debré, Paris, France; 3 Université de Paris, Epidémiologie Clinique et Évaluation Économique Appliquées aux Populations Vulnérables (ECEVE), Inserm, Paris, France; 4 Inserm, Epidémiologie Clinique et Évaluation Économique Appliquées aux Populations Vulnérables (ECEVE), U1123, Paris, France; 5 Inserm, Centre d'Investigation Clinique (CIC) 1426, Paris, France; 6 Centre de Référence des Maladies Endocriniennes Rares de la Croissance, Department of Pediatric Endocrinology and Diabetology, Assistance Publique-Hôpitaux de Paris, Hôpital Robert Debré, Paris, France; 7 Inserm, NeuroDiderot, Université de Paris, Paris, France

## Abstract

**Importance:**

In health care systems in which hospital reimbursement is based on a national mean length of stay (LOS), disadvantaged patients with an increased LOS may be a source of inefficiency. This implication has been reported in adult patients, but pediatric data have been scarce.

**Objective:**

To examine the association of patient socioeconomic status with hospital efficiency and financial balance in pediatrics.

**Design, Setting, and Participants:**

This cohort study obtained data from the French national hospital discharge database covering a 3-year period, from January 1, 2012, to December 31, 2014. Statistical analyses were performed between June 2016 and December 2018. All inpatient stays in hospital pediatric wards in mainland France by children older than 28 days or younger than 18 years (n = 4 121 187) were included. Admissions with coding errors or missing values for social disadvantage and/or cost calculations were excluded.

**Exposure:**

Social disadvantage was estimated with an ecological indicator, the FDep, available at the patient’s postcode of residence and divided into national quintiles.

**Main Outcomes and Measures:**

Efficiency was assessed through the variations in patient LOS compared with different national mean LOS (for pediatric patients, pediatric patients with a similar condition, and pediatric patients with a similar condition and severity level). Hospital financial balance was assessed at the admission level through the ratio of production costs to revenues and at the hospital level through the difference between aggregated revenues and production costs. Multivariate regression models examined the association between these indicators and socioeconomic status.

**Results:**

A total of 4 121 187 admissions were included (2 336 540 [56.7%] male; mean [SD] age, 7.4 [5.8] years). In all, 1 561 219 patients (37.9%) were in the 2 most disadvantaged quintiles. Patient LOS was shorter than the national mean LOS (mean [SD], 1.73 [4.21] days) for patients in the least disadvantaged quintile and longer for those in the more disadvantaged quintile (mean [SD], 1.67 [4.33] days vs 1.82 [4.14] days). This difference was higher for diagnosis related groups that included both adult and pediatric patients (mean [SD], 1.46 [4.22] days vs 1.61 [4.13] days) compared with those dedicated to pediatric patients (2.22 [4.13] days vs 2.12 [4.53] days). Patients in the most disadvantaged quintile were associated with a 3.2% increase in LOS (odds ratio, 1.0322; 95% CI, 1.0302-1.0341) compared with the national mean LOS. Social disadvantage was also associated with a significant increase in financial deficit for hospitals with 20% to 60% of patients in the 2 most disadvantaged quintiles (estimate: −€146 389; 95% CI, −€279 566 to −€13 213).

**Conclusions and Relevance:**

Patient socioeconomic status appears to be statistically significantly associated with an increase in LOS and cost in French hospitals with pediatric departments. This finding suggests that initiating reform in hospital payment methods may improve resource allocation efficiency and equity in access to pediatric care.

## Key Points

**Question:**

Is a pediatric patient’s socioeconomic status associated with the admitting hospital’s efficiency and financial balance?

**Findings:**

In this cohort study of 4 121 187 pediatric admissions in France, the socioeconomic status of the child was statistically significantly associated with increased length of stay and cost. A disadvantaged case mix was also statistically significantly associated with the financial balance of the hospital.

**Meaning:**

These findings suggest that reform of hospital funding to better consider patients’ socioeconomic status and the specificities of pediatric care should be encouraged in France and similar countries that rely on a reimbursement system based on diagnosis related groups.

## Teaser

This cohort study examines the association of pediatric inpatient socioeconomic status with the hospital’s efficiency and financial balance in France.

## Introduction

Since the 1990s, most developed health care systems in the western world have moved from a per diem payment system (in which hospitals are paid a given amount for each inpatient day) to a diagnosis related group (DRG) system (in which at least part of acute care hospital funding is activity based).^1,2,3^ The DRG system relies on a comprehensive patient classification system: an algorithm based on patient diagnoses, procedures, age, and other variables classifies each admission into a single DRG, which must be coherent both clinically and in terms of resource use. In this way, length of stay (LOS) no longer plays the most important role in hospital funding. Hospitals also may be encouraged to decrease LOS to create additional revenue because they are paid a given amount or charge regardless of LOS,^4,5,6^ whereas an LOS that is greater than the national mean on which the DRG charge is based may incur negative repercussions on its efficiency. Hospitals with highly disadvantaged catchment areas may therefore face challenges, given that social disadvantage has been shown to be associated with an increased LOS in adult patients.^7,8,9,10,11,12^ Yet these findings are rarely, if ever, taken into account in DRGs. As a consequence, disadvantaged patients could have major implications for hospital efficiency and financial balance.

This association could be especially true for children, as physicians may be more reluctant to discharge pediatric patients if their living environment is problematic or if their parents’ lack of understanding renders follow-up care challenging. Studies have shown that children from socioeconomically disadvantaged families are more often admitted in neonatal^13,14^ and pediatric^15,16^ wards, but few studies have focused on LOS. Studies that did evaluate LOS found conflicting results^17,18,19,20,21^ and were often limited by their sample size or population, with focus on a single disease or setting. In particular, 2 studies on bronchiolitis and 1 study on the 4 most common infectious diseases in pediatrics found no association between social disadvantage and LOS,^17,18,19^ and neither did research on children admitted for any reason in 2 National Health Service hospitals.^21^ On the other hand, a national study conducted in Taiwan found a significant association between total LOS for potentially preventable hospitalizations during the first 2 years of life and family income for some of the hospitalizations under study.^20^ However, to our knowledge, no study has ever gone a step further and assessed whether increased LOS was correlated with hospitals’ financial balance.

An additional challenge for pediatric patients is that they rarely have dedicated DRGs. Instead, they are often included in adult DRGs, which may have further repercussions on a hospital’s financial balance if the charges do not correspond to pediatric resource use.

These considerations hold especially true for the French health care system, in which hospital charges along with possible charges for daily supplements are set yearly at the national level by the statutory health insurance (SHI); charges are based on the annual production cost study of a sample of voluntary hospitals. Although French hospitals may be further compensated at a daily rate for admissions with extremely long LOS, most are reimbursed the same amount regardless of patient LOS. Consequently, hospitals with many patients with longer LOS will be negatively affected. Therefore, the objective of this study was to examine the association between patient socioeconomic status and hospital efficiency among all children admitted to hospitals with pediatric wards in mainland France and to assess the budgetary implications of caring for these patients for hospitals’ financial balance.

## Methods

### Study Design and Data Sources

This cohort study was conducted in France over a 3-year period, from January 1, 2012, to December 31, 2014, using a national administrative database. Access to the database was granted by the French Data Protection Authority, and the ethics committee of the Robert Debré Hospital approved the study. No informed consent was obtained because the study used anonymized patient data. This report followed the Strengthening the Reporting of Observational Studies in Epidemiology (STROBE) reporting guideline.

The cohort was extracted from the acute care hospital discharge database. This unique database of the SHI was created for hospital payment purposes, providing information on all hospitalizations in acute care hospitals.^22^ Patients are anonymized with a national identification number, and each admission is coded using DRGs. Variables in the database include patient characteristics (eg, age, sex, and postcode of residence), details of the admission (eg, principal and secondary diagnoses, length of stay, mode of admission and discharge, and severity level), and some information on health care consumption while hospitalized (eg, number of daily supplements for intensive care, surgical procedures).

Data from the annual national production cost study were used to calculate production costs for each admission.^23^ This study on voluntary hospitals throughout France provides detailed information on DRG costs and statistics (such as mean LOS) and serves as a basis for DRG charges. It is routinely used in French economic evaluations of hospital production costs.^24,25,26^ Cost data from the SHI were the basis for calculating hospital revenue using DRG charges and daily supplement charges as well as other modifiers.^27^

### Study Population and Measure of Socioeconomic Status

Included in the study were children older than 28 days (to exclude the neonatal period) and younger than 18 years (the legal limit for pediatric care in France) discharged between January 1, 2012, and December 31, 2014, from inpatient stays in hospitals with a pediatric ward throughout mainland France. The neonatal period was excluded from the analysis because it has major specificities in age and reason for admission. Admissions with coding errors (n = 1 448 309) or missing values for social disadvantage and/or cost calculations, including admissions not reimbursed through DRG charges (n = 62 800), were excluded from the analysis.

No individual socioeconomic measure exists in the discharge database; therefore, an ecological index of deprivation, the FDep, was used as a proxy.^28^ As other ecological indicators (such as the Carstairs or the Townsend indices^29,30^) against which it has been validated,^28^ the FDep is calculated according to 4 variables measured at the patient postcode of residence: percentage of blue-collar workers in the labor force, percentage of high school graduates in the population aged 15 years or older, unemployment rate in the labor force, and median income per household. The FDep was developed specifically for France and is routinely divided into quintiles using a national value set as a reference, with the lower quintile corresponding to the least disadvantaged population.

### Indicators of Efficiency at Patient Admission Level

The association between patient socioeconomic status and hospital efficiency was assessed through the variation in patient LOS in comparison with national mean LOS. To learn whether patient LOS increased with social disadvantage, we compared a patient’s LOS with the mean LOS of all included patients regardless of clinical condition by dividing patient LOS by the mean LOS in the study population.

Because case mix may vary with social disadvantage, we adjusted the variation in LOS by clinical condition by comparing patient LOS with the mean LOS of all admissions for a similar condition. We calculated the mean LOS of each DRG’s root (excluding the last number, which accounts for severity) and divided each patient LOS by the mean LOS of the patient’s corresponding DRG root. Furthermore, we adjusted the variation in LOS by patient severity within their own clinical condition. We calculated the mean LOS of each DRG (including severity level) and divided each patient LOS by the mean LOS of the patient’s DRG.

In addition, we calculated readmission rates at 15 days to ensure that an early discharge (and a short LOS) was not associated with increased readmission rates. We selected 15-day readmissions because early readmissions have been shown to be associated with the care received during the previous admission or follow-up rather than with the occurrence of other diseases or the natural course of the initial disease.^31,32,33^ Readmissions were identified through record linkage in the database.

### Indicators of Financial Balance at Patient and Hospital Admission Levels

The financial balance at the admission level was measured by calculating production costs and revenues for each admission and then comparing them to assess whether each admission production cost was higher than the reimbursement to the hospital. Production costs were calculated by adjusting the DRG’s mean production cost from the national production cost study for the year of discharge to the patient’s own LOS. Revenues were calculated using the SHI charges under the all-payer perspective (including the SHI, complementary health insurance, and out-of-pocket expenses).

Given that charges are based on DRGs’ mean LOS in hospitals participating in the national production cost study, which may differ from the true national mean LOS depending on the case mix of the hospitals, we compared patients’ LOS to their DRG’s LOS in the national production cost study to learn whether social disadvantage was associated with an increase in the ratio.

To identify whether socioeconomic status was associated with the financial balance of hospitals, we subtracted revenues and production costs for each admission and aggregated the results for each hospital using its identification number. Only hospitals with a minimum of 100 admissions over the 3-year period were included in the analysis because we assumed that hospitals with fewer admissions were likely to have changed status during the study period and to no longer have pediatric wards.

### Statistical Analysis

Characteristics of the population were described using mean (SD) for continuous variables, except for costs described with median (interquartile range [IQR]), or number (percentage) for the categorical variables. Variables included patient characteristics (eg, age, sex, and quintile of social disadvantage) and admission characteristics (eg, admission through the emergency department, severity level). Severity was pooled into 3 categories: nonsevere, intermediate, and severe. Any admission with an intensive care unit stay was classified as severe. No statistical test was performed because the study used exhaustive data.

Indicators of efficiency and financial balance were described in a similar manner for the whole population and for each quintile of social disadvantage. Readmissions at 15 days were standardized on the severity of the initial admission so as not to introduce bias.

Multivariate regression models were carried out to assess the association between social disadvantage and our indicators at a patient admission level after a log transformation. This association is illustrated here with the model of the variation of patient LOS in comparison to the national mean pediatric LOS to present results adjusted for clinical condition and severity. To account for the nested structure of the data,^34^ we ran multilevel models with 2 levels: admission and hospital. Admission characteristics included patient age, sex, quintile of social disadvantage, severity, type of admission (medical, surgical, or interventional), major diagnostic category (eg, neurology, urology, dermatology), and environmental characteristics (eg, available medical care). Hospital characteristics included case-mix data (eg, mean age, percentage of disadvantaged patients), structural characteristics (eg, number of beds, teaching status), and environmental characteristics (eg, presence of housing facilities for families with financial difficulties). To confirm the existence of a random effect at the hospital level, we first ran a null model without any explanatory variables (model 1). We then introduced patient characteristics (model 2) and added variables calculated at the hospital level (model 3).

A multivariate generalized linear model was carried out to assess whether a disadvantaged case mix was associated with hospital annual mean financial balance after adjusting for the percentage of disadvantaged patients in its case mix as well as other case-mix, structural, and environmental characteristics. Explanatory variables were included in the multivariate model if they were associated with the dependent variable at the statistical level of 2-sided *P* = .20 in the univariate analysis.

All analyses were performed between June 2016 and December 2018. SAS software, version 9.4 (SAS Institute Inc) was used.

## Results

### Characteristics of the Population

A total of 5 632 296 inpatient pediatric admissions were identified between January 1, 2012, and December 31, 2014, in hospitals with a pediatric department located in mainland France. Of this total, 4 121 187 admissions (73.2%), distributed among 1100 hospitals, met the inclusion criteria (Figure).

**Figure.**
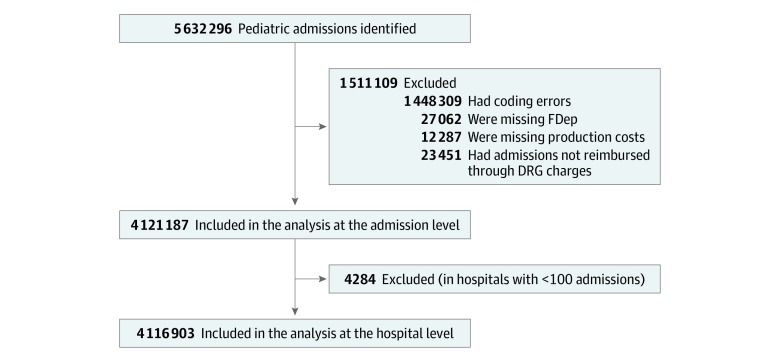
Flowchart of Pediatric Admissions in the Study DRG indicates diagnosis related group; FDep, ecological index of deprivation.

Male patients represented the majority of the population (2 336 540 [56.7%]). The mean (SD) age of patients was 7.4 (5.8) years. The distribution of inpatient stays along the social disadvantage gradient was close to that of the French population (1 561 219 [37.9%] in the 2 most disadvantaged quintiles; Table 1). Most admissions were in a mixed (adult and pediatric) DRG. Patients in the most disadvantaged quintile, compared with patients in the least disadvantaged quintile, were more likely to have intermediate (285 149 [34.0%] vs 298 346 [28.6%]) or severe (33 419 [4.0%] vs 40 827 [3.9%]) admissions (Table 1) and were admitted through the emergency department more often (322 719 [38.5%] vs 336 391 [32.3%]). No difference in hospital mortality was observed between the patients in different quintiles (Table 1).

**Table 1.** Characteristics of the Admissions by Quintile of Social Disadvantage

Variable	No. (%)					
	FDep Quintile					Total
	1 (Least Disadvantaged)	2	3	4	5 (Most Disadvantaged)	
Admissions	1 042 717 (25.3)	854 163 (20.7)	663 088 (16.1)	722 028 (17.5)	839 191 (20.4)	4 121 187
Age, mean (SD), y	7.6 (5.8)	7.4 (5.8)	7.5 (5.8)	7.3 (5.7)	7.0 (5.7)	7.4 (5.8)
Male	597 524 (57.3)	483 620 (56.6)	372 364 (56.2)	408 175 (56.5)	474 857 (56.6)	2 336 540 (56.7)
Severity						
Nonsevere	703 544 (67.5)	557 213 (65.2)	423 324 (63.8)	460 755 (63.8)	520 623 (62.0)	2 665 459 (64.7)
Intermediate	298 346 (28.6)	265 043 (31.0)	215 851 (32.6)	234 950 (32.5)	285 149 (34.0)	1 299 339 (31.5)
Severe	40 827 (3.9)	31 907 (3.7)	23 913 (3.6)	26 323 (3.7)	33 419 (4.0)	156 389 (3.8)
Admission through the ED	336 391 (32.3)	298 293 (34.9)	243 767 (36.8)	261 202 (36.2)	322 719 (38.5)	1 462 372 (35.5)
Mixed DRG	721 721 (69.2)	576 058 (67.4)	442 057 (66.7)	477 938 (66.2)	548 724 (65.4)	1 354 689 (67.1)
In-hospital death	869 (0.08)	716 (0.08)	541 (0.08)	606 (0.08)	752 (0.09)	3484 (0.08)

Abbreviations: DRG, diagnosis related group; ED, emergency department; FDep, ecological index of deprivation.

Excluded patients were similar to included patients in sex and percentage in the 2 most disadvantaged quintiles (41.1% after excluding patients with a missing FDep), but excluded patients were older (mean [SD] age of 10.2 [4.9] years).

### Association Between Socioeconomic Status and Efficiency at Patient Admission Level

Mean LOS was close to 10% higher in patients in the most disadvantaged quintile compared with those in the least disadvantaged quintile (mean [SD], 1.82 [4.14] days vs 1.67 [4.33] days; Table 2). Similar results were found between patients in the most and least disadvantaged quintiles when accounting for patient clinical condition (mean [SD] ratio, 1.0294 [1.20] vs 0.9774 [1.14]) and for both clinical condition and severity (mean [SD] ratio, 1.0109 [0.56] vs 0.9892 [0.52]), although the difference between patients in the least and most disadvantaged quintiles grew smaller with each adjustment, indicating an important role of patient case mix in the association between socioeconomic status and efficiency. After adjusting for the severity of the initial admission, the readmission rate at 15 days was similar for all quintiles of social disadvantage (Table 2).

**Table 2.** Association of Socioeconomic Status With Hospital Efficiency and Financial Balance at Patient Admission Level

Variable	FDep Quintile					Total Population
	1 (Least Disadvantaged)	2	3	4	5 (Most Disadvantaged)	
**Indicators of Hospital Efficiency, Mean (SD)**						
LOS, d	1.67 (4.33)	1.71 (4.28)	1.72 (4.08)	1.76 (4.18)	1.82 (4.14)	1.73 (4.21)
Ratio of patient LOS						
vs National LOS	0.9626 (2.50)	0.9870 (2.47)	0.9937 (2.36)	1.0172 (2.41)	1.0502 (2.39)	1 (2.43)
After adjusting for clinical condition	0.9774 (1.14)	0.9922 (1.21)	0.9993 (1.26)	1.0082 (1.22)	1.0294 (1.20)	1 (1.20)
After adjusting for clinical condition and severity	0.9892 (0.52)	0.9973 (0.50)	1.0022 (0.59)	1.0041 (0.52)	1.0109 (0.56)	1 (0.54)
Readmissions at 15 d, %^a^	7.56	7.54	7.45	7.55	7.59	NA
**Indicators of Hospital Financial Balance, Median (IQR)**						
Production costs, €^b^	1034 (723)	1055 (733)	1071 (747)	1056 (759)	1056 (877)	1055 (749)
Revenues, €	932 (820)	940 (847)	941 (845)	940 (860)	940 (877)	940 (850)
Ratio of production costs vs revenues	1.0785 (0.54)	1.0856 (0.56)	1.0916 (0.57)	1.0933 (0.57)	1.0996 (0.57)	1.0880 (1.09)
Ratio of patient LOS vs national production cost study LOS^c^	1.2812 (1.78)	1.2550 (1.60)	1.2391 (1.64)	1.2401 (1.50)	1.2340 (1.73)	1.2520 (1.67)

Abbreviations: FDep, ecological index of deprivation; IQR, interquartile range; LOS, length of stay; NA, not applicable.

^a^ Standardized on the severity of the initial admission.

^b^ To convert to US$, multiply by 1.10272.

^c^ The national production cost study is an annual study of voluntary hospitals that provides detailed information on diagnosis related group costs and statistics (such as mean LOS) and then serves as a basis for calculating diagnosis related group charges.

The difference between patients in the least and most disadvantaged quintiles was higher for mixed DRGs (9.8% higher; mean [SD], 1.46 [4.22] days vs 1.61 [4.13] days) compared with pediatric-only DRGs (4.5% higher; 2.22 [4.13] days vs 2.12 [4.53] days), when comparing patient LOS with the national LOS (mean [SD], 1.73 [4.21] days) (eTable in the Supplement).

In the multivariate multilevel regression model, we found that an increase in the quintile of social disadvantage was statistically significantly associated with an increase in patient LOS compared with the national mean LOS (3.2% higher in patients in the most disadvantaged quintile compared with those in the least disadvantaged quintile; odds ratio [OR], 1.0322; 95% CI, 1.0302-1.0341; Table 3). Overall, this increase added up to 40 670 extra hospital days annually for patients not in the least disadvantaged quintile. Being female (OR, 1.0417; 95% CI, 1.0405-1.0428), being in a mixed DRG (OR, 1.0188; 95% CI, 1.0174-1.0202), or having intermediate admissions (OR, 3.2668; 95% CI, 3.2625-3.2713) or severe admissions (OR, 8.2334; 95% CI, 8.2087-8.2573), compared with nonsevere admissions, were also significantly associated with an increase in the ratio. Increase in age (OR, 1.0007; 95% CI, 1.0006-1.0008) and availability of general practitioners (OR, 1.0002; 95% CI, 1.0002-1.0002) were significantly associated with the higher ratio, although the OR was very small. All major diagnostic categories introduced in the model were significantly associated with the ratio.

**Table 3.** Factors Associated With the Variation in Patient Length of Stay vs National Length of Stay^a^

Variable	Odds Ratio (95% CI)
Intercept	0.2745 (0.2565-0.2937)
**Patient Social Disadvantage**	
Socioeconomic status by quintiles	
Q1 (least disadvantaged)	1 [Reference]
Q2	1.0158 (1.0141-1.0176)
Q3	1.0203 (1.0184-1.0222)
Q4	1.0263 (1.0244-1.0282)
Q5 (most disadvantaged)	1.0322 (1.0302-1.0341)
**Other Patient Characteristics**	
Mean age	1.0007 (1.0006-1.0008)
Sex	
Male	1 [Reference]
Female	1.0417 (1.0405-1.0428)
Severity of the admission	
Nonsevere	1 [Reference]
Intermediate	3.2668 (3.2625-3.2713)
Severe	8.2334 (8.2087-8.2573)
Type of DRG	
Pediatric	1 [Reference]
Mixed	1.0188 (1.0174-1.0202)
Type of admission	
Medical	1 [Reference]
Surgical	0.9017 (0.9001-0.9032)
Interventional	0.8542 (0.8522-0.8561)
Main diagnostic category	
Central nervous system	1 [Reference]
Ophthalmology	1.1283 (1.1227-1.1339)
Ear, nose, and throat	1.2471 (1.2437-1.2504)
Pulmonology	1.6112 (1.6069-1.6156)
Circulatory system	1.4041 (1.3979-1.4104)
Gastroenterology	1.6606 (1.6563-1.6648)
Musculoskeletal apparatus	1.3037 (1.2999-1.3075)
Dermatology	1.1972 (1.1934-1.2012)
Endocrinology	1.4588 (1.4538-1.4636)
Urology	1.2537 (1.2501-1.2573)
Gynecology	1.7828 (1.7716-1.7941)
Infant-related illnesses	9.990 (9.0178-9.3849)
Hematology	1.4715 (1.4659-1.4771)
Infectious diseases	1.0132 (1.0088-1.0176)
Mental disorders	1.3007 (1.2959-1.3056)
Trauma, allergies, and poisoning	1.3850 (1.3792-1.3908)
Unclassified illnesses	1.3800 (1.3758-1.3843)
Organ transplant	8.3612 (8.1442-8.5840)
Availability of general practitioners in town^b^	1.0002 (1.0002-1.0002)
**Hospital Case-Mix Characteristics**	
Mean age	0.9974 (0.9943-1.0006)
Girls, %	1.0000 (0.9991-1.0009)
Patients in the 2 most disadvantaged quintiles, %	
<20	1 [Reference]
20-60	0.9961 (0.9863-1.0060)
>60	1.0002 (0.9894-1.0111)
Admissions, %	
Severe	1.0000 (0.9988-1.0011)
With mixed DRG	
<60	1 [Reference]
60-85	0.9520 (0.9367-0.9675)
≥85	0.9554 (0.9366-0.9746)
Surgical	0.9994 (0.9989-0.9999)
Interventional	0.9997 (0.9992-1.0002)
Central nervous system	1.0007 (0.9991-1.0022)
Pulmonology	1.0040 (1.0020-1.0059)
Gastroenterology	0.9976 (0.9970-0.9982)
Dermatology	0.9996 (0.9987-1.0005)
Endocrinology	0.9975 (0.9957-0.9993)
Urology	0.9998 (0.9993-1.0004)
Infant-related	0.9986 (0.9735-1.0242)
Infectious diseases	1.0012 (0.9964-1.0060)
Mental disorders	1.0025 (1.0009-1.0040)
Trauma, allergies, and poisoning	0.9992 (0.9972-1.0012)
Unclassified illnesses	0.9968 (0.9960-0.9977)
**Hospital Environmental Characteristics**	
Teaching hospital	
No	1 [Reference]
Yes	1.0134 (1.0037-1.0231)
No. of pediatric beds	1.0001 (1.0001-1.0001)
Admissions with 15-d readmissions, %	1.0040 (1.0031-1.0048)
Housing facilities near the hospital	
No	1 [Reference]
Yes	1.0044 (1.0005-1.0083)

Abbreviations: DRG, diagnosis related group; Q, quintile.

^a^ Intraclass coefficient = 22.20% in the null model, 3.00% after introducing patient variables, and 1.31% after introducing patient and hospital variables.

^b^ Availability derived from an indicator that measures the spatial adequation between supply and demand, based on distance, activity of the professionals to account for part-time-only activity, and age of the population to account for need.

Compared with medical admissions, surgical (OR, 0.9017; 95% CI, 0.9001-0.9032) and interventional (OR, 0.8542; 95% CI, 0.8522-0.8561) admissions were associated with a decrease in LOS. At the hospital level, characteristics that were statistically significantly associated with the ratio were mainly structural and case mix–related variables. The percentage of patients in the 2 most disadvantaged quintiles in the hospital case mix was not associated with LOS.

### Association Between Socioeconomic Status and Financial Balance at Patient Admission and Hospital Levels

Median (IQR) production costs were higher in the most disadvantaged quintile (€1034 [€723]; to convert to US$, multiply by 1.10272), whereas median (IQR) revenues were similar in the least disadvantaged (€932 [€820]) and most disadvantaged (€940 [€87]) quintiles (Table 2). The higher production costs led to more than €7.5 million in extra costs for patients not in the least disadvantaged quintile compared with those in the least disadvantaged quintile. The median ratio also increased with each quintile in the descriptive analysis. In addition, when comparing patient LOS to the mean LOS of their DRG in the national production cost study, we found that patients in the most disadvantaged quintile had a smaller ratio compared with patients in the least disadvantaged quintile (1.2340 [1.73] vs 1.2812 [1.78]), which is inconsistent with our findings on mean DRG LOS in the database (Table 2). This finding may be associated with the presence of mixed DRG (eTable in the Supplement).

In general, pediatric admissions were a source of deficit in activity-based payments for 1029 hospitals (93.6%). Over the 3-year period, the mean (SD) total deficit was €814 441 (€2 923 654), with a mean (SD) annual deficit of €290 150 (€1 054 073). The total deficit itself appeared smaller in hospitals with few disadvantaged patients but grew as the percentage of disadvantaged patients increased before decreasing again (eFigure in the Supplement).

In the multivariate regression model, a similar trend was found: having 20% to 60% of patients in the 2 most disadvantaged quintiles was associated with a statistically significant increase in hospital deficit compared with hospitals with less than 20% disadvantaged patients (estimate: −€146 389; 95% CI, −€279 566 to −€13 213), and no association was found when the percentage of these patients was greater than 60% (estimate: €21 858; 95% CI, −€125 908 to €169 623; Table 4). Other characteristics were associated with increased mean annual deficit, including the mean annual number of pediatric admissions (estimate: −€125; 95% CI, −€140 to −€109) and some case-mix characteristics, such as a percentage of mixed DRGs greater than 60% and the percentage of patients admitted for certain types of diagnosis (ie, central nervous system diseases, respiratory tract disorders, endocrine disorders, or trauma). Moreover, higher percentages of severe admissions (estimate: €19 406; 95% CI, €2391-€36 420) or admissions through the emergency department (estimate: €6230; 95% CI, €1182-€11 279) were associated with lower mean annual deficit.

**Table 4.** Factors Associated With Mean Annual Financial Balance

Variable	Estimate (95% CI), €
Intercept	160 919 (−1 001 409 to 1 323 247)
**Social Disadvantage in the Hospital Case Mix**	
Patients in the 2 most disadvantaged quintiles, %	
<20	1 [Reference]
20-60	−146 389 (−279 566 to −13 213)
≥60	21 858 (−125 908 to 169 623)
**Other Case-Mix Characteristics**	
Mean age	23 828 (−26 351 to 74 007)
Girls, %	3728 (−10 704 to 18 160)
Admissions, %	
Severe admissions	19 406 (2391 to 36 420)
Through the ED	6230 (1182 to 11 279)
With mixed DRG	
<60	1 [Reference]
60-85	−568 643 (−808 920 to −328 365)
≥85	−436 890 (−720 148 to −153 633)
Surgical	−827 (−8415 to 6761)
Interventional	−3500 (−12 449 to 5450)
Central nervous system	−40 379 (−66 363 to −14 395)
Ear, nose, and throat	2473 (−3133 to 8078)
Pulmonology	−36 627 (−65 978 to −7276)
Gastroenterology	−486 (−10 923 to 9951)
Dermatology	6190 (−7503 to 19 884)
Endocrinology	−37 183 (−72 441 to −1925)
Urology	3618 (−4712 to 11 948)
Infant-related	−52 056 (−673 432 to 569 319)
Hematology	−766 (−18 219 to 16 687)
Infectious diseases	47 581 (−21 643 to 116 805)
Mental disorders	931 (−21 725 to 23 587)
Trauma, allergies, and poisoning	−46 673 (−86 808 to −6537)
Unclassified illnesses	803 (−11 052 to 12 657)
**Hospital Environmental Characteristics**	
Teaching hospital	
No	1 [Reference]
Yes	26 063 (−131 051 to 183 176)
Mean annual No. of pediatric admissions	−125 (−140 to −109)
Housing facilities near the hospital	
No	1 [Reference]
Yes	−120 608 (−248 852 to 7636)

Abbreviations: DRG, diagnosis related group; ED, emergency department.

## Discussion

Patient socioeconomic status was statistically significantly associated with an increase in LOS and cost. Overall, this increase corresponded to up to 40 670 extra hospital days annually, costing €7 847 967 for patients not in the least disadvantaged quintile compared with those in the least disadvantaged quintile. Socioeconomic status was also significantly associated with hospitals’ financial balance, especially those with 20% to 60% of pediatric patients in the 2 most disadvantaged quintiles. Pediatric admissions were a source of deficit for hospitals, with most hospitals in the study experiencing a deficit. The DRGs covering both adult and pediatric clinical conditions were associated with an increase of that deficit.

A possible explanation for the deficit associated with pediatric activity is that most French DRGs are not specific to pediatric patients and that more than 60% of pediatric admissions are sorted into DRGs that include both adults and children despite children requiring greater use of resources and being underrepresented in the annual national production cost study.

In addition, the reduced association with social disadvantage when adjusting for clinical condition and severity is likely because of variations in case mix. More than 2082 separate DRGs are included in this analysis, and the results show some heterogeneity in the association with major diagnostic categories. Further analyses of main diagnoses are ongoing to identify those variations.

The absence of an association between hospital financial balance and case mixes with more than 60% of patients in the most disadvantaged quintiles could hint at adaptation strategies put in place by hospitals with highly disadvantaged catchment areas to care for these patients given the hospitals’ available budget. Further studies are needed to understand these mechanisms and the potential repercussions on patients’ quality of care.

Because the national production cost study is carried out on a set of voluntary hospitals, they may have fewer disadvantaged patients than represented in the national mean, which would explain the discrepancy in our findings when comparing patient LOS with the mean LOS of their DRG (a greater ratio in disadvantaged patients when we use the true national LOS of their DRG and a greater ratio in advantaged patients when we use the LOS of their DRG in the national production cost study). A better representation of participating hospitals should therefore be encouraged so that charges may be closer to the true mean production costs.

To our knowledge, no country currently uses patient socioeconomic status as a determinant for charges; they could therefore gain an advantage from investigating whether they should use this factor. We think this finding is of particular importance to ensure that hospitals covering disadvantaged populations provide the best care to their patients without being penalized or practicing *cream skimming* (defined as choosing patients for some characteristics other than their need for care and excluding others [in this case, disadvantaged patients] to enhance profitability).

Based on the findings, 2 strategies have emerged to take into account the higher costs incurred by disadvantaged patients. First, a modulation of DRG charges using an allocation key (either global or specific to each clinical situation) at the patient level could be considered to help mitigate the implication of social disadvantage for hospitals in countries in which it has not yet been implemented. Second, given the association between DRGs that include both adult and pediatric patients and both efficiency and financial balance, we believe DRGs specific to children should become the norm whenever possible in all DRG-based systems to provide an adequate picture of the resources used during pediatric admission and therefore an appropriate charge.

Such measures to reform hospital payment methods could serve as an example for other, similar DRG-based health care systems and we believe should be encouraged where applicable to improve resource allocation efficiency and equity in access to pediatric care.

### Strengths and Limitations

To our knowledge, this study is the first to look at the association between social disadvantage and hospital efficiency and financial balance in a pediatric population at the national level without focusing on specific conditions, such as infectious diseases,^15,17,18,19,35^ diabetes,^36,37,38^ and preterm or low-birth-weight infants,^14,39,40,41^ or on a small number of hospitals or local area.^16,18,20,21^ This study also relied on a vast administrative database, which allowed the exhaustive and comprehensive representation of the entire French pediatric population admitted to hospitals.

However, this study also has limitations. Our analysis was restricted to the variables included in the discharge database, which was built for reimbursement purposes and not research. We had to use an ecological proxy for patient socioeconomic status, derived from patients’ postcode of residence, which could not take into account extreme situations such as homelessness. In addition, the database had no indicators of clinical behaviors. Although the 3-year study period was relatively short and should allow for homogeneous clinical practices, we could not exclude a change in behaviors during that time.

Moreover, the study period is now 5 years in the past, partly owing to regulatory delays associated with gaining access to the database. However, we do not believe this dating affected the validity of the results, as no major changes have occurred in how DRG charges are calculated in the past 5 years.

The large sample size of this study must lead us to question statistical significance vs real-life relevance, as other studies have pointed out in the past.^42,43,44^ Although we found many significant associations, some were so small that they are unlikely to have implications for everyday practice. However, those variables were only used as adjustment variables, and the rate ratios associated with social disadvantage seem to imply real-life relevance.

## Conclusions

This study found that patient socioeconomic status appears to be significantly associated with an increase in LOS and cost in French hospitals with pediatric departments. The findings highlight the need to reform hospital funding to better take into account patient socioeconomic status and the specificities of pediatric care in France and possibly other countries that use a DRG-based reimbursement system.

## Notes

Supplement. **eTable.** Association Between Socioeconomic Status and Hospital Efficiency and Financial Balance at a Patient’s Admission Level by Type of DRG **eFigure.** Mean Annual Financial Balance of Hospitals With Regards to Their Pediatric Admissions Depending on Their Percentage of Disadvantaged Patients
