# Supplementary material for: Association of Pediatric Inpatient Socioeconomic Status With Hospital Efficiency and Financial Balance
Source: JAMA Netw Open. 2019 Oct 18;2(10):e1913656. doi: 10.1001/jamanetworkopen.2019.13656 (PMC6813670; doi:10.1001/jamanetworkopen.2019.13656)
Supplement: Supplement. — eTable. Association Between Socioeconomic Status and Hospital Efficiency and Financial Balance at a Patient’s Admission Level by Type of DRG eFigure. Mean Annual Financial Balance of Hospitals With Regards to Their Pediatric Admissions Depending on Their Percentage of Disadvantaged Patients [file jamanetwopen-2-e1913656-s001.pdf]

Supplementary Online Content

Michel M, Alberti C, Carel J-C, Chevreul K. Association of pediatric inpatient socioeconomic status with hospital efficiency and financial balance. *JAMA Netw Open*. 2019;2(10):e1913656. doi:10.1001/jamanetworkopen.2019.13656

eTable. Association Between Socioeconomic Status and Hospital Efficiency and Financial Balance at a Patient's Admission Level by Type of DRG

eFigure. Mean Annual Financial Balance of Hospitals With Regards to Their Pediatric Admissions Depending on Their Percentage of Disadvantaged Patients

This supplementary material has been provided by the authors to give readers additional information about their work.

© 2019 Michel M et al. *JAMA Network Open*

eTable. Association Between Socioeconomic Status and Hospital Efficiency and Financial Balance at a Patient's Admission Level by Type of DRG

	FDep quintile 1 (least disadvantaged)	FDep quintile 2	FDep quintile 3	FDep quintile 4	FDep quintile 5 (most disadvantaged)	Total population
Indicators of hospital efficiency						
Mean length of stay, in days (SD)						
Pediatric DRG	2.12 (4.53)	2.13 (4.37)	2.11 (4.24)	2.17 (4.58)	2.22 (4.13)	2.15 (4.38)
Mixed DRG	1.46 (4.22)	1.51 (4.22)	1.53 (3.98)	1.55 (3.94)	1.61 (4.13)	1.53 (4.11)
Mean ratio of patient LOS vs. national LOS (SD)						
Pediatric DRG	1.2246 (2.62)	1.2310 (2.52)	1.2189 (2.45)	1.2555 (2.64)	1.2801 (2.38)	1.2424 (2.53)
Mixed DRG	0.8456 (2.44)	0.8693 (2.43)	0.8811 (2.30)	0.8955 (2.27)	0.9286 (2.38)	0.8813 (2.38)
Mean ratio after adjusting for clinical condition (SD)						
Pediatric DRG	0.9782 (1.30)	0.9901 (1.25)	0.9912 (1.28)	1.0110 (1.33)	1.0310 (1.22)	1 (1.28)
Mixed DRG	0.9771 (1.06)	0.9932 (1.19)	1.0034 (1.25)	1.0068 (1.15)	1.0286 (1.19)	1 (1.16)
Mean ratio after adjusting for clinical condition and severity (SD)						
Pediatric DRG	0.9870 (0.55)	0.9975 (0.54)	1.0000 (0.56)	1.0056 (0.56)	1.0121 (0.59)	1 (0.56)
Mixed DRG	0.9901 (0.51)	0.9972 (0.49)	1.0033 (0.61)	1.0034 (0.49)	1.0103 (0.54)	1 (0.52)
Indicators of hospital financial balance						
Median production costs, in € (IQR)						
Pediatric DRG	1,147 (902)	1,222 (850)	1,222 (850)	1,234 (928)	1,250 (1,036)	1,222 (892)
Mixed DRG	968 (656)	968 (656)	990 (656)	968 (656)	960 (656)	968 (656)
Median revenues, in € (IQR)						
Pediatric DRG	1,073 (1,014)	1,091 (1,002)	1,091 (985)	1,091 (999)	1,168 (1,033)	1,091 (1,006)
Mixed DRG	859 (649)	859 (674)	892 (672)	859 (678)	859 (682)	859 (678)

© 2019 Michel M et al. *JAMA Network Open*

Median ratio of production costs vs. revenues (IQR)						
Pediatric DRG	1.0268 (0.61)	1.0598 (0.64)	1.0695 (0.64)	1.0695 (0.63)	1.0695 (0.63)	1.0598 (0.64)
Mixed DRG	1.1310 (0.50)	1.1343 (0.51)	1.1445 (0.51)	1.1608 (0.53)	1.1687 (0.53)	1.1440 (0.51)
Mean ratio of patient LOS vs. national production cost study's LOS (SD)						
Pediatric DRG	1.0837 (1.08)	1.0889 (0.72)	1.0878 (0.79)	1.0922 (0.77)	1.1002 (0.95)	1.0904 (0.81)
Mixed DRG	1.3691 (2.08)	1.3352 (1.88)	1.3149 (1.93)	1.3156 (1.76)	1.3049 (2.02)	1.3314 (1.95)

© 2019 Michel M et al. *JAMA Network Open*

eFigure. Mean Annual Financial Balance of Hospitals With Regards to Their Pediatric Admissions Depending on Their Percentage of Disadvantaged Patients

x-axis: percentage of patients in a hospital's two most disadvantaged quintiles

y-axis: mean annual financial balance

n: number of hospitals in the corresponding category

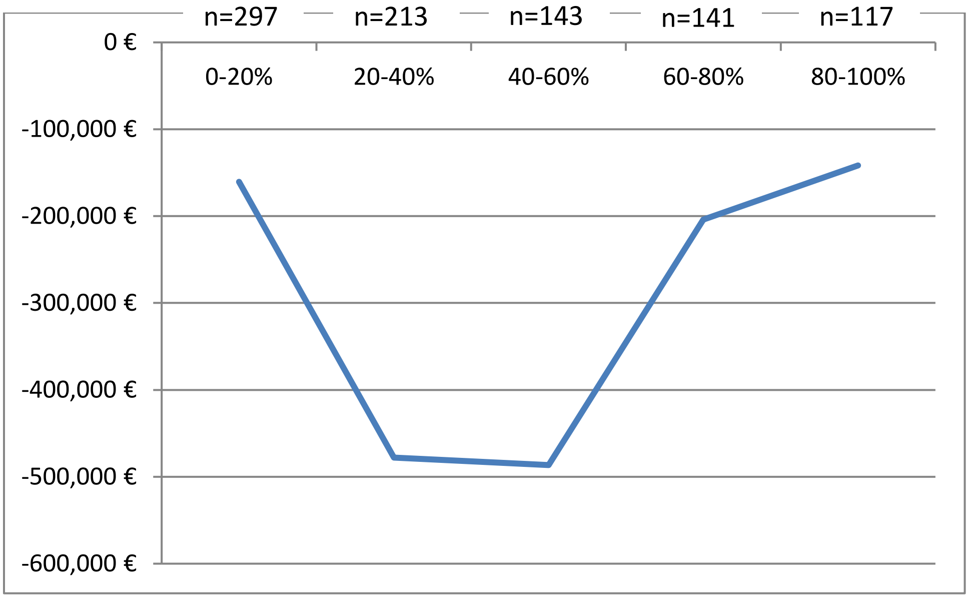

© 2019 Michel M et al. *JAMA Network Open*
